# Diagnostic Accuracy of PET/CT-Guided Percutaneous Biopsies for Malignant Peripheral Nerve Sheath Tumors in Neurofibromatosis Type 1 Patients

**DOI:** 10.1371/journal.pone.0138386

**Published:** 2015-10-07

**Authors:** Mehdi Brahmi, Philippe Thiesse, Dominique Ranchere, Thomas Mognetti, Stephane Pinson, Caroline Renard, Anne-Valérie Decouvelaere, Jean-Yves Blay, Patrick Combemale

**Affiliations:** 1 Centre Léon Bérard, Lyon, France; 2 Edouard Herriot Hospital Genetic Laboratory, Lyon, France; BIDMC, UNITED STATES

## Abstract

**Background:**

Malignant peripheral nerve sheath tumors (MPNST) are one of the most frequent causes of death in patients with neurofibromatosis type 1 (NF1). Early detection is crucial because complete surgical resection is the only curative treatment. It has been previously reported that an ^18^F-fluorodeoxyglucose (FDG) positron emission tomography/computed tomography (PET/CT) image with a T/L (Tumor/Liver) SUV_max_ ratio > 1.5 provides a high negative predictive value; however, it is not specific enough to make a NF1-related MPNST diagnosis. A formal proof of malignant transformation from a histological analysis is necessary before surgical excision because the procedure can cause mutilation. The objective of the present work was to investigate the effectiveness of and complications associated with PET/CT-guided percutaneous biopsies for an NF1-related MPNST diagnosis.

**Methods:**

PET/CT-guided percutaneous biopsy procedures performed on 26 NF1 patients with a clinical suspicion of MPNST and a suspect lesion from a PET/CT scan (T/L SUV_max_ ratio > 1.5) were retrospectively evaluated. The localization of the suspected malignant site was determined using PET/CT. A stereotactic (ultrasonic and CT control) core biopsy technique was used with a local anesthesia.

**Results:**

The first PET/CT-guided percutaneous biopsies enabled a pathological diagnosis for all of the patients (no "inconclusive " results were obtained), and no secondary procedures were needed. Among the 26 patients, the histopathological results from the biopsy were malignant in 17 cases and benign (BPNST with atypical cells) in nine cases. No complications from the diagnostic procedure were observed. A surgical resection was performed in 18 patients (seven benign and 11 malignant biopsies), removing the fine needle biopsy scar. In addition, six locally advanced/metastatic MPNST were treated with chemo/radiotherapy, and two BPNST had no progression after a follow-up of 14 and 39 months, respectively. The PET/CT-guided percutaneous biopsy gave 25 accurate diagnoses and one false negative (BPNST with atypical cells on the biopsy and MPNST on the operated tumor), resulting in a diagnostic accuracy rate of 96%. This false negative case may be explained by the high heterogeneity of the tumor: benign areas were contiguous with the malignant ones and associated with inflammation.

**Conclusions:**

PET/CT-guided percutaneous biopsies are an effective and relatively non-traumatic procedure for diagnosis of NF1-related MPNST. It is the most reliable approach for early detection of MPNST.

## Introduction

Neurofibromatosis type 1 (NF1) is the most common neurogenetic disorder (1:2500 have this disease) [1]. It predisposes the affected individuals to the development of benign peripheral nerve sheath tumors (BPNST), which may undergo a malignant transformation (malignant peripheral nerve sheath tumors; MPNST) [2]. MPNST are one of the most frequent causes of death in NF1 patients [3], and the risk of MPNST is estimated to be 8–13% over their lifetime [2,4]. Early diagnosis of MPNST is crucial because complete surgical resection is the only curative treatment. However, it is often difficult to distinguish between BPNST and MPNST purely on the basis of clinical symptoms. Computed tomography (CT) or magnetic resonance imaging (MRI) and a formal proof of malignant transformation are necessary to make a definitive diagnosis and before performing a possibly mutilating excision.

Currently, ^18^F-fluorodeoxyglucose (FDG) positron emission tomography/computed tomography (PET/CT) is the most efficient imaging modality for detection of MPNST in NF1 patients [5]. Combemale et al. previously reported that a 1.5 cut-off for the tumor/liver binding ratio (T/L SUV_max_ ratio) provides a 98.8% negative predictive value (NPV). However, this T/L SUV_max_ ratio can produce false positives, resulting in a 61.5% positive predictive value (PPV). Therefore, PET/CT imaging alone is not specific enough to diagnose NF1-related MPNST, and a histopathological analysis remains necessary for an optimal diagnosis and the subsequent decision making about the condition.

Percutaneous biopsies to detect malignancy are technically challenging in these patients. We hypothesized that using PET/CT imaging for guidance might facilitate a successful biopsy and diagnosis of MPNST in NF1 patients. To the best of our knowledge, this combination has never been investigated. We conducted the present study to evaluate the diagnostic accuracy, suitability, and complications associated with PET/CT-guided percutaneous biopsies for the diagnosis of NF1-related MPNST.

## Materials and Methods

This retrospective monocentric study was conducted between October 2003 and November 2014 at the regional competence center for neurofibromatosis in Centre Leon Berard (Lyon, France). NF1 patients with a clinical suspicion of MPNST and a suspect lesion found from PET/CT underwent PET/CT-guided percutaneous biopsies, the standard practice for diagnostic, and a histopathological diagnosis was performed. The inclusion criteria for this study were fulfillment of the National Institutes of Health diagnostic criteria for NF1 (Table 1), clinical symptoms suggesting MPNST (pain, neurological deficit, and/or enlargement of a pre-existing BPNST), a T/L SUV_max_ ratio ≥ 1.5 from the PET/CT images, and percutaneous biopsies with available histopathological analysis. The exclusion criteria were a T/L SUV_max_ ratio < 1.5, lack of percutaneous biopsy, patient refusal, or the patient was under palliative care.

**Table 1:** Consensus criteria for the diagnosis of neurofibromatosis type 1*.

NIH criteria for the diagnosis of neurofibromatosis type 1	
**1**	Six or more cafe-au-lait skin macules >5 mm in prepubertal individuals and >15 mm in postpubertal individuals
**2**	Two or more neurofibromas of any type or one plexiform neurofibroma
**3**	Axillary or inguinal freckling
**4**	Two or more Lisch nodules
**5**	Optic glioma
**6**	Bone lesion with sphenoid dysplasia or thinning of the long bone cortex with or without pseudarthrosis
**7**	A first-degree relative (parent, sibling, or offspring) that meets NIH criteria

*The diagnosis of NF1 requires at least two of the seven NIH criteria.

The study was performed according to the French laws at the time of the initiation of the study and followed the principles laid down in the Declaration of Helsinki. Oral consent was obtained after a complete explanation to the patients or legal representatives for minors and it was reported in the patient's medical record. As the study was not interventional (standard of practice for diagnostic), formal written consent was not required by French law. The Centre Léon Bérard Clinical Trial Review Committee (Institutionnal Review Board of the institution) reviewed and agreed with the study protocol and this consent procedure. The completed STARD checklist [6] is provided as S1 Checklist.

### Biopsy preparation

Before the percutaneous biopsy procedure, patients were informed about the purpose and methodology of the technique and the possible complications. Bleeding time parameters were measured before the biopsy to ensure that they were within the normal limits. The localization of the supposed transformed area (MPNST) was determined using PET/CT. The shortest and safest tissue distance to reach the lesion was determined. The distance of the lesion from the entry point and the entry angle were planned. One hour before the procedure, patients were given an analgesic (morphine) and anxiolytic.

### Biopsy procedure

**Fig 1:**
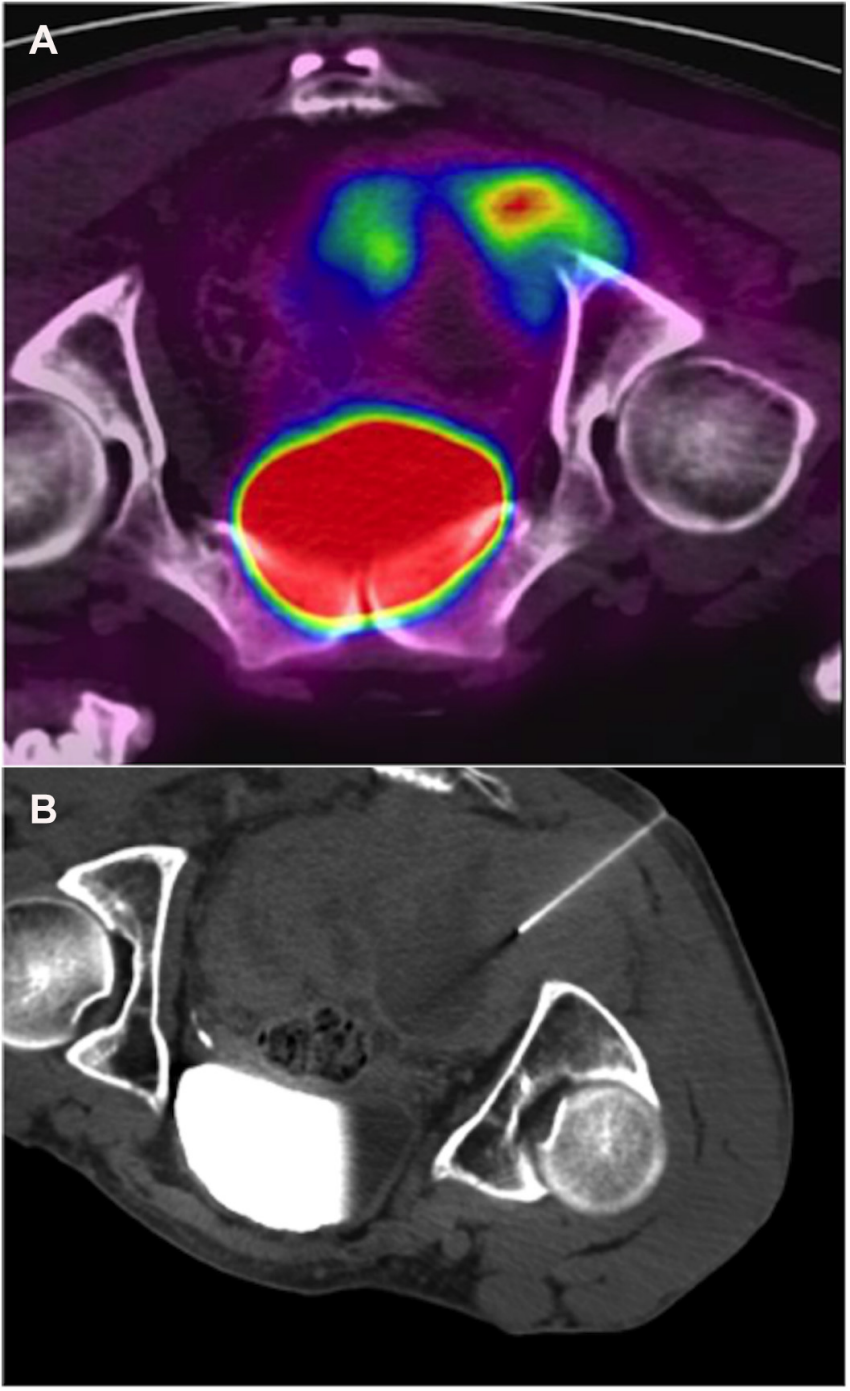
PET/CT-guided percutaneous biopsy of a metabolically active pelvic soft-tissue tumor in 34-year-old man with NF1. (a) Initial PET/CT discriminate FDG-avid portion at the right sciatic notch. (b) The needle is shown to be in most metabolically active portion of FDG-avid mass. Pathology results concluded to intermediate-grade MPNST. The tumor was unresectable and the patient was treated by chemotherapy (adriamycin and ifosfamide). He died 17 months after the diagnosis.

Inhalation sedation with a 50% nitrous oxide/oxygen premix (Kalinox™) was given to all of the children and those adults whose lesion was painful. Under strict aseptic conditions, a local anesthesia of lidocaine was used to numb the skin and subcutaneous tissues. A short incision (< 10 mm) was made, and a 17-G coaxial needle guide was advanced into the skin, using ultrasonic and CT control sections to ensure the proper position of the needle (Fig 1). Two fine needle aspirations (22 G) were taken for cytological analyses. Next, an 18-G biopsy pistol was placed inside the needle guide and multiple (4–10) biopsies, using 18-G Tru Cut^®^ biopsy needles, were performed. The fine needle biopsy scar was tattooed in anticipation of a surgical resection. The samples were placed into a 4% formalin solution. At the end of the procedure, patients were transferred for room observation. For one patient, percutaneous access was not possible because of the deep location of the tumor (retrorectal); a laparotomy was performed to obtain the biopsy.

### Pathology results

The tumors were diagnosed and classified by the Department of Pathology at the Centre Leon Bérard (Lyon), one of the pathology reference centers for sarcomas in France (www.rreps.org). A cytological analysis was performed on the fine needle aspirations. Light microscopy and immunohistochemical studies (including panCK AE1/AE3, EMA, CD34, SMA, Desmin, PS 100, and Ki67) were used to analyze the formalin-fixed paraffin-embedded tissues from the percutaneous biopsies. Diagnoses about the malignancy of the tumors (MPNST) were based on the light microscopic features, such as increased cell density with a change in architecture, mitotic activity, atypical scattered cells with nuclear polymorphism, and necrosis. The histological grading and tumor differentiation were determined according to the updated FNCLCC system.

### Statistical analysis

Statistics were performed using SPSS statistical software (version 15.0 for Windows, SPSS, Inc). The diagnostic accuracy of the technique was assessed in terms of sensitivity, specificity and positive and negative predictive value (PPV; NPV).

## Results

A total of 26 patients with NF1 (15 women and 11 men) were included in this retrospective study (Table 2). All of the patients were treated at the Centre Leon Berard. The median age was 30 years old (range 16–64), and the median lesion size was 65 mm (range 30–154).

**Table 2:** Baseline characteristics of patients.

N	26
**Median age**	30 (16–64)
**Median lesion size (mm)**	65 (30–154)
**Median T/L SUV**_**max**_ **ratio**	2.7 (1.6–3.3)
**Biopsy pathology results**	
Malignant	17
Benign	9
Non diagnostic (Inconclusive result)	0

The PET/CT-guided percutaneous biopsies, which were performed first, enabled a pathological diagnosis for all of the patients (no “inconclusive” results were obtained). A second procedure was not necessary. Histopathological analyses of the biopsies revealed 17 MPNST and 9 BPNST; the BPNST had insufficient nuclear atypia to categorize them as MPNST. A surgical resection was performed in 18 patients, removing the fine needle biopsy scar (marked by Chinese ink). Among the eight patients who did not undergo surgery, six had locally advanced (unresectable) or metastatic MPNST and were treated with chemotherapy or radiotherapy. The other two patients had BPNST: no tumor progression was observed during the follow-up (14 and 39 months, respectively), supporting their diagnosis as benign tumors.

The PET/CT-guided percutaneous biopsies provided 25 accurate diagnoses and one false negative diagnosis (BPNST with nuclear atypia from the histopathological analysis of the biopsy and MPNST from the histopathological analysis of the resected tumor), resulting in a diagnostic accuracy rate of 96% (*n* = 25/26). The sensitivity, specificity, PPV and NPV for the diagnosis of MPNST were 94%, 100%, 100% and 89%, respectively.

**Fig 2:**
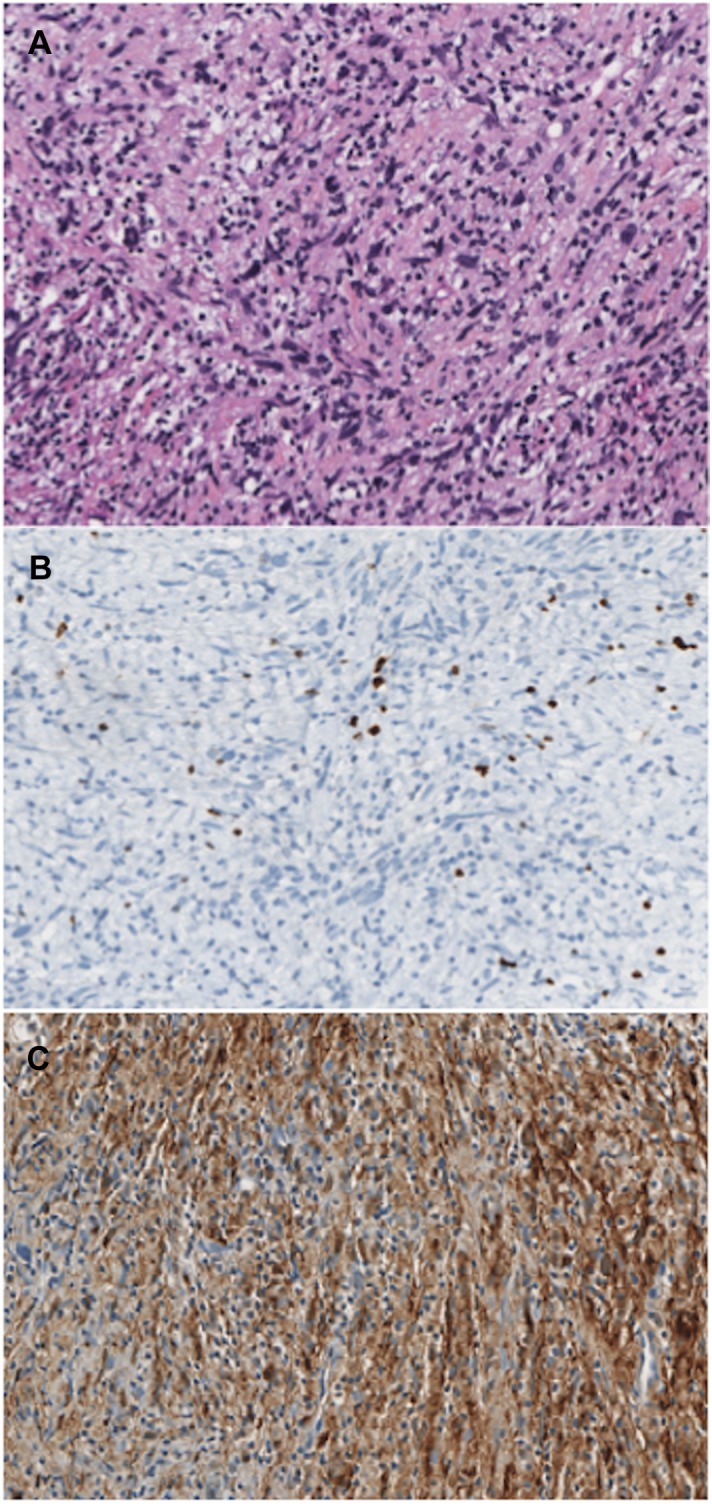
Histopathological results of the biopsy of the false negative case. A. Atypical scattered cells with enlarged and hyperchromatic nuclei, in the absence of mitotic figures. B. Low Ki67 index (≈ 2%). C. Immunohistochemical staining for S100 is strongly positive.

**Fig 3:**
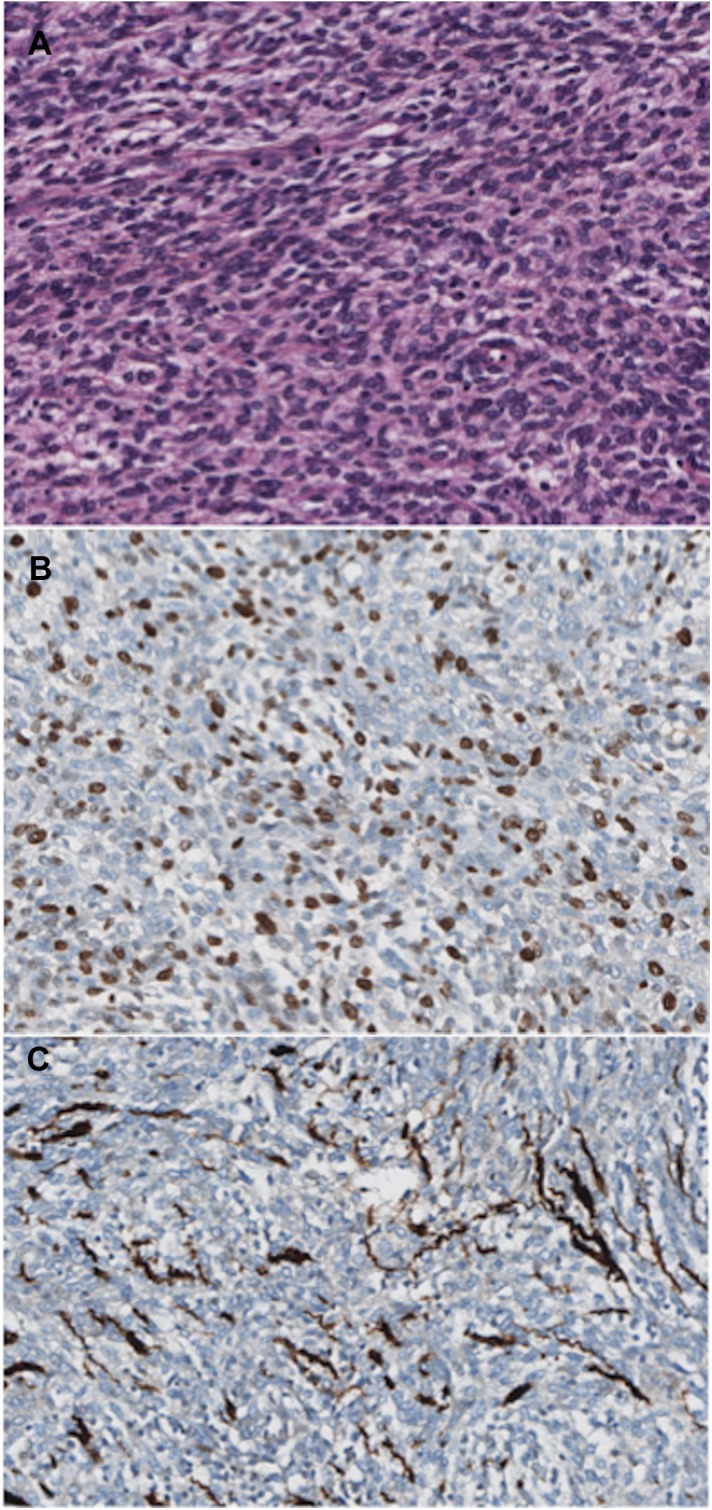
Histopathological results of the resected tumor of the false negative case. A. The neoplastic cells in this area are cohesive. The cells have eosinophilic cytoplasm and nuclei with prominent nucleoli. Several mitoses are present. B. High Ki67 index (≈ 50%). C. Immunohistochemical staining for S100 is focal.

The histopathological analysis of the false negative biopsy (Fig 2) showed atypical scattered cells, with increased size and hyperchromatic nuclei associated with inflammation. However, mitoses, increased cell density, and necroses were not detected. The Ki67 index was low (≈ 2%), and the S100 immunohistochemical staining was strongly positive. Therefore, it was categorized as a “BPSNT with atypical cells” (and not MPNST). After surgical resection, the histopathological analysis (Fig 3) showed a highly heterogeneous tumor: benign areas were contiguous with the malignant ones (with a maximum tumor mitotic rate of eight mitoses per 10 HPF, a high Ki67 index of about 50%, and focal S100 immunohistochemical staining) and associated with a dense peritumoral lymphocytic infiltrate (stromal reaction).

No complications were reported during or after the percutaneous biopsies. The local anesthesia was often sedation inhalation with Kalinox^TM^ (*n* = 12/25, 48%). Hospitalization was not required, except for one patient with a deep retrorectal tumor who underwent a laparotomy under general anesthesia.

The median follow-up was 10 months for the NF1 patients with a diagnosis of MPNST (range 3–71) and 25 months for the surviving patients (range 2–71). At the time of the last follow-up, 12 of the 18 patients (67%) had died from the disease, and 6 of the 18 patients (33%) were alive: two patients (11%) had no evidence of the disease, and four (22%) still had the disease. Local progression along the biopsy site was not observed during the follow-up.

For the patients who underwent a resection of the MPNST (*n* = 12/18, 67%), the median relapse-free survival (time from surgery to recurrence) was seven months (range 3–46). For all of the patients with an MPNST, the median overall survival was nine months (range 2–71).

## Discussion

To the best of our knowledge, this is the first study to present and analyze the diagnostic strategy of combining PET/CT imaging with percutaneous biopsies for MPNST detection. We observed a high diagnostic accuracy rate (96%), confirming the value of PET/CT-guided percutaneous biopsies as an effective tool for detecting NF1-related MPNST. This technique was performed in an outpatient setting, using sedation inhalation by Kalinox^TM^ as the local anesthesia, except for one deep retrorectal tumor. No percutaneous biopsy-related complications were reported. Our data indicate that this diagnosis strategy is safe, effective, feasible, and comfortable for patients.

NF1-related MPNST are associated with a poor prognosis [7], especially when late diagnoses are made [8]. Therefore, an effective diagnosis strategy is necessary. The clinical symptoms of MPNST include pain (PPV 67% and NPV 75%), neurological deficits (PPV 57% and NPV 91%), and enlargement of a pre-existing PNST in NF1 patients (PPV 95% and NPV 92%) [9]. However, these symptoms are not specific to malignant transformation [7]. CT and MRI images can detect malignant transformation of soft tissue tumors. Indeed, many criteria have been proposed for the diagnosis of malignant soft-tissue lesions using MRI [10–12]. Van Herendael et al. [13] reported factors significantly associated with MPNST: intramuscular distribution (60% sensitivity and 79% specificity), location along a large nerve (57% sensitivity and 99% specificity), and nodular morphology (97% sensitivity and 23% specificity). While MRI provides useful information, these criteria are not specific or sensitive enough to make a definitive diagnosis. Moreover, the presence of multiple tumors on a given site makes an MRI assessment even more difficult.

Several studies have analyzed the use of PET/CT in NF1-related MPNST diagnoses; the high variability in SUV_max_ cut-off values was proposed as a means to distinguish between MPNST and BPNST [14–20]. However, false positives were always present, and the high variability of the SUV_max_ cut-off values prevented this distinction. Chirindel et al. [21] tried to improve the diagnostic performance using a late acquisition protocol (four hour versus one hour); however, there were no significant benefits for differentiating MPNST from BPNST. Urban et al. [22] evaluated the ability of a whole-body MRI and FDG-PET fusion to improve diagnosis. This combination seemed to help guide the decision about whether to biopsy lesions that were suggestive of malignant transformation. However, the study was limited by the small number of tissue biopsies available and its inability to correlate the functional, morphological imaging characteristics with the histopathological results. The value of using PET for detecting NF1-associated MPNST was confirmed by our group [5], where we revealed that a T/L SUV_max_ ratio < 1.5 could eliminate malignant transformation, with a 98.8% NPV. When the T/L ratio was ≥ 1.5, there was a strong likelihood of malignancy, with a 61.5% PPV. Unfortunately, this method also resulted in a significant number of false positives, and a histopathological confirmation is recommended before any potentially detrimental therapeutic decisions are made. Indeed, the treatment of MPNST requires a large and often mutilating resection, which differs from the treatment of BPNST, where a marginal excision is acceptable. These results highlight the necessity for a minimally invasive biopsy before a surgical resection. This can be achieved with PET/CT-guided percutaneous biopsies.

The current method using PET/CT-guided percutaneous biopsies is the best approach for an NF1-related MPNST diagnosis and for assessing the type of surgery necessary for treatment. The percutaneous biopsies were both safe and feasible to perform in most of the tumor locations (in our cohort, n = 25/26, 96%), with the one exception of a patient with a deep retrorectal tumor. This diagnosis strategy provided only a single false negative (BPNST with atypical cells on the biopsy and MPNST on the resected tumor). This false negative was a result of the high heterogeneity of this particular tumor and its inflammatory component. Furthermore, this tumor was one of the larger ones of the cohort (120 mm). For larger tumors, it may be necessary to increase the number of percutaneous biopsies. Moreover, molecular analysis of those biopsies might be helpful. Using array comparative genomic hybridization and mutation analysis, Beert et al. [23] identified several genetic alterations in MPNST that were not present in BPNST with atypia, especially mutations in CDKN2A and TP53. However, they were unable to genetically distinguish BPNST with atypia from low-grade MPNST. Finally, the tumor from the false negative patient was resected when it later increased in size and became painful, highlighting the importance of clinical symptoms when a malignant transformation is suspected. Diagnosis of NF1-related MPNST should be based on converging criteria, including clinical symptoms, imaging, histopathology, and, at times, tumor genotype.

Interestingly, the histopathological analyses of the eight BPNST (which showed a T/L SUV_max_ ratio ≥ 1.5) always revealed the presence of atypical scattered cells, with enlarged and hyperchromatic nuclei, but without any other changes, such as increased cellularity, mitoses, or necroses. These so-called “borderline BPNST”, which also showed an increased glucose uptake on the PET/CT [15], may be a transition state between BPNST and MPNST [23–25]. Because of their potential role in MPNST development, the identification and monitoring of patients with these precursor lesions may facilitate an early diagnosis of MPNST and improvement in prognosis. For the patients diagnosed with BPNST with nuclear atypia who did not undergo a surgical resection, two of the patients were stable at the median follow-up of 36 months. Currently, there are no recommendations about BPNST with atypical cells. For a localized tumor, a preventive, non-mutilating surgical resection could be performed. However, for a large tumor that requires a mutilating excision, a second percutaneous biopsy or close monitoring with PET/CT should be done.

Because of the retrospective design of this study and relatively small number of patients, a larger prospective study is necessary to confirm the value of the combination of PET/CT and percutaneous biopsies to diagnose MPNST.

In conclusion, PET/CT-guided percutaneous biopsies provide a rapid, relatively non-traumatic, and effective procedure for diagnosing NF1-related MPNST. This combination is the most reliable approach for early detection of MPNST, reducing the need for more invasive surgical procedures.

## Supporting Information

S1 Checklist STARD Checklist. (DOC)
